# Prenatal Detection and Neonatal Management of Tuberous Sclerosis Complex Compounded by Symptomatic Cardiac Arrhythmia

**DOI:** 10.1155/crpe/1952300

**Published:** 2026-06-19

**Authors:** Raja Imad Abu Iram, Abdulrahman Hussein Karaki, Hisham Dweik, Mohammad I. Smerat

**Affiliations:** ^1^ College of Medicine and Health Sciences, Palestine Polytechnic University, Hebron, State of Palestine, ppu.edu; ^2^ Department of Pediatric Cardiology, Al-Ahli Hospital, Hebron, State of Palestine, ahlihospital.com; ^3^ Department of Radiology, Al-Ahli Hospital, Hebron, State of Palestine, ahlihospital.com

**Keywords:** case report, cortical tubers, hamartoma, rhabdomyoma, subcortical tubers, subependymal nodules, tuberous sclerosis, tuberous sclerosis complex (TSC)

## Abstract

Tuberous sclerosis complex (TSC) is a rare autosomal dominant syndrome affecting multiple organ systems through the development of benign hamartomas. We present a newborn female patient suspected antenatally of having TSC based on fetal echocardiography, which suggested cardiac rhabdomyomas; these findings were confirmed postnatally. The patient was managed with regular multidisciplinary follow‐up and treated according to her presenting manifestations, including neonatal cardiac arrhythmias. This case highlights the importance of early genetic confirmation and proactive management of neonatal complications.

## 1. Introduction

Tuberous sclerosis, also called tuberous sclerosis complex (TSC), is a rare, multisystemic syndrome with autosomal dominant inheritance, characterized by tumorigenesis that can affect almost every organ in the body, most commonly the brain, heart, skin, kidney, and lungs [1].

The first documented case of TSC was described by the German pathologist von Recklinghausen in 1862. It was in a baby with sclerotic brain lesions and cardiac myotomes, who did not live long after birth. The pathogenesis of TSC is driven by de novo or inherited mutations in the TSC1 gene on Chromosome 9, or the TSC2 gene on Chromosome 16 [2].

Advancements in imaging have allowed for the detection of cardiac rhabdomyomas in fetuses during the prenatal period via ultrasound (US) screening. While US has limited sensitivity for evaluating fetal brain lesions, MRI is highly reliable in detecting the extent of brain involvement [3].

Early diagnosis is critical as it allows for the implementation of targeted surveillance and management strategies. In this report, we describe a newborn female with TSC confirmed via whole exome sequencing (WES) after prenatal findings of multiple intracardiac masses and neonatal brain lesions.

## 2. Case Presentation

This female patient is the third child of nonconsanguineous Palestinian parents. She was delivered at 37 weeks of gestation via cesarean section due to cardiac concerns identified by prenatal fetal echocardiography, which revealed multiple intracardiac masses in both ventricles. The family history was significant for an affected maternal half‐brother previously diagnosed with TSC. At birth, the patient weighed 3200 g, with APGAR scores of 6 at 1 min and 9 at 5 min. Shortly after delivery, she developed secondary apnea requiring positive pressure ventilation (bagging), after which she was admitted to the neonatal intensive care unit (NICU).

The patient was generally afebrile and appeared pink. A septic workup was performed with unremarkable findings and negative cultures. The initial neonatal neurological examination noted axial hypotonia. Brain MRI demonstrated cortical and subcortical tubers (Figure 1) and subependymal nodules (SENs) (Figure 2).

**FIGURE 1:**
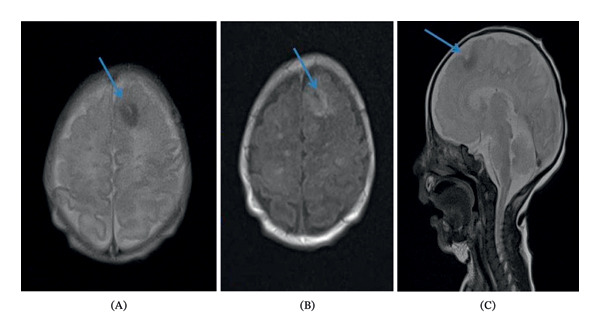
Axial views of FLAIR (A) and T1 (B) and sagittal view of T2 (C) show left frontal cortical lesion (blue arrows).

**FIGURE 2:**
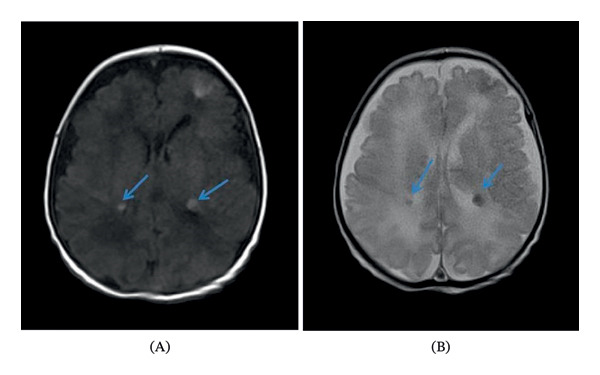
Axial views of T1 (A) and T2 (B) show subependymal nodules (blue arrows).

Cardiovascular evaluation revealed frequent bradycardia without murmurs. A 24‐h Holter monitor identified frequent ventricular and supraventricular extrasystoles (premature contractions), though no sustained ventricular or supraventricular tachycardia was recorded. Postnatal echocardiography confirmed multiple intracardiac masses in the left ventricle, right ventricle, and right atrium (Figure 3).

**FIGURE 3:**
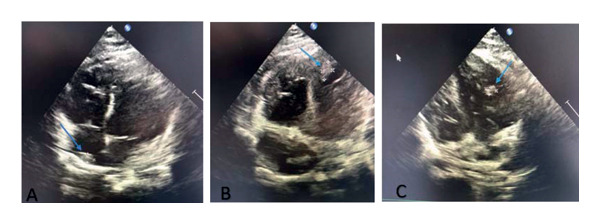
Multiple intracardiac masses. A: RA mass. B: LV mass. C: RV mass.

Based on these findings, the patient met the 2021 International Tuberous Sclerosis Complex Consensus Group criteria for a “definite” diagnosis, having three major features: cortical dysplasias, SENs, and cardiac rhabdomyomas. Genetic confirmation was achieved via WES, which identified a heterozygous pathogenic variant in the TSC2 gene (TSC2: c.1832G > A, p.Arg611Gln).

## 3. Discussion

TSC is characterized by the overactivation of the mechanistic target of rapamycin (mTOR) pathway due to mutations in the TSC1 or TSC2 tumor suppressor genes [4]. While approximately one‐third of cases are inherited, the majority occur de novo [5]. The phenotypic expression is highly variable, involving benign hamartomas in the skin, brain, heart, lungs, kidneys, and eyes [6, 7].

Cardiac rhabdomyomas are the most common primary heart tumors in fetuses and children [8]. Although these tumors often regress spontaneously, they can cause significant neonatal morbidity if they obstruct blood flow or disrupt the electrical conduction system [9]. In our case, the presence of multiple rhabdomyomas was likely associated with the observed bradycardia and frequent extrasystoles. To manage these arrhythmias, the patient was started on oral amiodarone (15 mg twice daily), highlighting the need for active pharmacological intervention in symptomatic neonates.

Regarding neurological involvement, the cortical tubers and SENs found in this patient require long‐term monitoring for the development of subependymal giant cell astrocytomas (SEGAs). Current guidelines recommend serial brain imaging every 1–3 years until Age 25 [10].

A literature review of 8 similar cases confirms that cortical dysplasia and cardiac rhabdomyomas are the most frequent findings in prenatally or neonatally diagnosed TSC (100% of cases reviewed). Our case adds to the growing body of evidence—including recent cohorts by Bakoš et al. [11] and Milon et al. [12]—emphasizing that multiple cardiac lesions are significantly more predictive of TSC than solitary tumors [11, 13]. Furthermore, early diagnosis provides an opportunity for potential prenatal interventions; for example, maternal administration of mTOR inhibitors has been shown to reduce the size of fetal rhabdomyomas in cases of hemodynamic instability [12].

The clinical utility of early diagnosis is further underscored by a comparative review of prenatally and neonatally detected TSC cases (Table 1). While historical literature focused heavily on the classic diagnostic triad—often absent in the neonatal period—our review of recent cohorts, including those described by Schwartz et al. [14] and Gow et al. [15], reveals a consistent correlation between multiple cardiac rhabdomyomas and early neurological involvement. Notably, 100% of the cases reviewed exhibited both cardiac and cortical lesions, aligning with our patient’s presentation. However, our case is distinguished by the severity of neonatal rhythm disturbances; whereas many reported rhabdomyomas remain asymptomatic, the frequent extrasystoles observed in this patient require active pharmacological intervention with amiodarone. This highlights the necessity of shifting the clinical focus from mere identification to proactive management of TSC‐related complications in the first days of life, as guided by the updated 2021 International TSC Consensus recommendations.

**TABLE 1:** Clinical characteristics, genetics, and management of prenatally diagnosed TSC cases.

First author	Mutation	Prenatal findings	Neonatal symptoms	Management/outcome
Present case	TSC2	Cardiac rhabdomyomas	Apnea, bradycardia, frequent extrasystoles	Started on amiodarone; stable on multidisciplinary follow‐up.
Bakoš [11]	Various	Multiple cardiac tumors	Arrhythmias, heart failure signs	Highlights higher TSC risk with multiple vs. solitary lesions.
Milon [12]	TSC1/TSC2	Cardiac rhabdomyomas (88%)	Neurological tubers, seizures	Retrospective study of 240 cases; emphasizes early MRI/Genetics.
Wortmann [16]	NA	Cardiac rhabdomyoma, SEGA	Respiratory distress	Surgical resection of SEGA; highlights severity of early brain lesions.
Fruitt [17]	NA	Multiple cardiac rhabdomyomas	signs of heart failure	Pharmacological management of hemodynamics; lesion regression noted.
Axt‐Fliedner [18]	NA	Cardiac masses, IUGR	Hemodynamic instability	Maternal corticosteroids; emphasis on prenatal monitoring of flow.
Chen [19]	NA	Cardiac rhabdomyoma, IUGR	Neonatal distress	Supportive care; focus on growth restriction in TSC fetuses.
Tworetzky [20]	NA	Intracardiac tumors	Conduction abnormalities	Found that 95% of fetuses with multiple tumors had TSC.

Abbreviation: NA = not available.

In conclusion, this case demonstrates that the prenatal detection of cardiac rhabdomyomas is a critical window for early TSC diagnosis and intervention. While these tumors are often benign, the potential for symptomatic neonatal arrhythmias requires immediate cardiovascular monitoring and the use of antiarrhythmic therapy when indicated. Adherence to the updated 2021 International TSC Consensus criteria and early clinical diagnosis remain the cornerstones of effective multidisciplinary management.

## Funding

No funding was received for this manuscript.

## Ethics Statement

Written informed consent was obtained from the parents of the patient for the publication of this case report and any accompanying images.

## Conflicts of Interest

The authors declare no conflicts of interest.

## Data Availability

The data that support the findings of this study are available from the corresponding author upon reasonable request.
